# The Variant T Allele of *SLC2A1* rs841847 Confers Moderate Protection Against Late-Onset Alzheimer’s Disease

**DOI:** 10.3390/biom16060808

**Published:** 2026-05-29

**Authors:** Ágnes Fehér, Anna Boldizsár, Magdolna Pákáski, Zoltán Janka, János Kálmán

**Affiliations:** 1 Department of Physiology, Albert Szent-Gyorgyi Medical School, University of Szeged, Dóm Tér 10, H-6720 Szeged, Hungary; 2 Department of Psychiatry, Albert Szent-Gyorgyi Medical School, University of Szeged, Korányi Fasor 8-10, H-6720 Szeged, Hungaryjanka.zoltan@med.u-szeged.hu (Z.J.); kalman.janos@med.u-szeged.hu (J.K.)

**Keywords:** Alzheimer’s disease, glucose transporter 1 (GLUT1), solute carrier family 2 member 1 gene (*SLC2A1*), apolipoprotein E gene (*APOE*), single nucleotide polymorphism (SNP), association study, rs841847

## Abstract

Epidemiological and biological evidence indicate a close connection between Alzheimer’s disease (AD) and type-2 diabetes mellitus. Glucose transporter 1 (GLUT1), encoded by the *SLC2A1* gene, has a major role in glucose metabolism, the dysregulation of which has been implicated in both diseases. We conducted a case-control association study in a sample of 439 non-diabetic patients with late-onset AD and 304 cognitively healthy, non-diabetic elderly controls to determine the potential risk for developing AD associated with *SLC2A1* rs841847 polymorphism. The rs841847 C/C genotype occurrence was higher in the AD group (AD: 60.4%, controls: 50.7%), while the minor T allele-containing genotypes were more frequent among controls (AD: 39.6%, controls: 49.3%). A multivariate logistic regression model adjusted for age, sex, and apolipoprotein E (*APOE*) ε4 status (ε4 allele carriers versus non-carriers) demonstrated that carriers of the T allele had a significantly reduced risk for AD compared to C/C homozygotes (OR = 0.672; 95% CI: 0.493–0.916; *p* = 0.012). Although the rs841847 polymorphism has been linked to type-2 diabetes mellitus, the present study investigated this gene variant in AD for the first time. Our findings indicate a moderate protective effect for the rs841847 T allele on the susceptibility to AD. We demonstrated the rs841847 polymorphism as a candidate single nucleotide polymorphism for further examination as a predisposing genetic factor for AD.

## 1. Introduction

Neurodegenerative late-onset Alzheimer’s disease (AD) is the most common form of dementia and represents a significant and growing public health problem worldwide [1]. AD is characterized by extracellular deposition of amyloid-β (Aβ) plaques, intracellular accumulation of hyperphosphorylated tau tangles, and consequent synaptic and neuronal loss, leading to cognitive and functional decline [2,3]. Late-onset AD is multifactorial and has a substantial heritability component; however, despite the extensive research, its complex genetic background is still not fully understood [4]. The largest effect locus, the apolipoprotein E gene (*APOE*; MIM# 107741), along with the 20 additional important loci identified by genome-wide association studies, explains approximately half of the estimated heritability [4,5].

Apolipoprotein E is a glycoprotein involved in cholesterol homeostasis and lipid metabolism, produced primarily by hepatocytes and astrocytes, and present in both plasma and cerebrospinal fluid [6]. The *APOE* gene is located on chromosome 19 at locus q13.31. Two common single nucleotide polymorphisms (SNPs) in exon 4 of the *APOE* gene (rs429358 and rs7412) define three haplotypes, commonly referred to as the ε2, ε3, and ε4 alleles, of which ε3 is the most prevalent globally (~75–80%), followed by ε4 (~10–15%) and ε2 (~5–10%). The rs429358 polymorphism (T > C) results in a cysteine to arginine amino acid change at position 112, while rs7412 (C > T) leads to an arginine to cysteine amino acid substitution at residue 158 [6]. Together, these two SNPs determine three common protein isoforms (ε2: Cys_112_, Cys_158_; ε3: Cys_112_, Arg_158_; ε4: Arg_112_, Arg_158_).

Apolipoprotein E modulates susceptibility to AD in an isoform-specific manner by influencing lipid transport and Aβ metabolism in the brain. The single amino acid substitutions in the different apolipoprotein E isoforms produce subtle structural differences that profoundly influence their binding affinities to lipids, Aβ, and cellular receptors [7]. The ε2, ε3, and ε4 variants modulate lipid composition of the neuronal membranes, which in turn influence the processing of amyloid-β precursor protein (AβPP) and the generation of aggregation-prone Aβ [8]. The ε4 isoform is associated with impaired Aβ clearance, as well as enhanced Aβ production and aggregation [9].

A large body of evidence has consistently identified the *APOE* ε4 allele as the strongest common genetic risk factor for late-onset AD. This association has been robustly replicated across diverse populations and ethnic groups. Numerous meta-analyses and large-scale genome-wide studies confirm its dose-dependent effect on disease risk [10,11]. Previous studies also consistently confirmed the role of *APOE* ε4 allele in AD in the Hungarian population [12,13,14,15,16,17].

Beyond the amyloid-cascade and tau hypotheses, which posit that the accumulation of the Aβ peptides and hyperphosphorylated tau proteins are the primary causative factors for developing AD, altered glucose metabolism and insulin resistance are also considered to play a major role in AD pathophysiology [18]. AD has even been proposed as a brain-specific type of diabetes [19]. The primary source of energy in the brain is glucose, which enters the brain across the blood–brain barrier via specialized transporters. Defects in cerebral glucose uptake and metabolism have been associated with AD, and this impairment seems to be a cause of neurodegeneration, rather than its consequence [20]. Accordingly, reductions in cerebral glucose metabolism have been correlated with worsened cognitive performance and faster cognitive decline in AD patients, further supporting the hypothesis that hypometabolic changes play a significant role in the pathogenesis of the disease [18].

The majority of glucose is transported to the brain via glucose transporter 1 (GLUT1), a membrane-bound transporter protein encoded by the solute carrier family 2 member 1 gene (*SLC2A1*; MIM#138140) [21]. In cerebral microvessels, brain glucose uptake correlated with GLUT1 levels [22,23]. Early reduction in glucose transport into the brain and reduced GLUT1 expression at the blood–brain barrier were reported in AD [24]. Reduced GLUT1 expression in amyloid-β precursor protein (AβPP) transgenic mice has been demonstrated to correlate with accelerated Aβ pathology and decreased Aβ clearance, profound changes in vascular, blood–brain barrier and neuronal function, including cerebral microvascular degeneration, blood–brain barrier breakdown, diminished neuronal activity, behavioral deficits, progressive neuronal loss and neurodegeneration [24].

Further support for the role of altered glucose homeostasis in AD comes from studies identifying blood-based biomarkers of AD. The membrane protein expression levels of the GLUT1 and the insulin receptor on the red blood cells were significantly elevated in AD patients as compared to age-matched healthy controls [25]. These findings on GLUT1 expression levels in AD at the blood–brain barrier versus on the erythrocytes suggest a tissue-specific alteration, and additional examinations are required to confirm these results and elucidate the underlying mechanisms. *APOE* ε4 allele is a major risk factor for AD and confers susceptibility to greater cognitive decline in the elderly [26]. The relationship between the *SLC2A1* gene and AD pathophysiology is also supported by the findings that *APOE* ε4 allele carriers displayed significant upregulation of transporters related to glucose homeostasis, including GLUT1 [27].

The *SLC2A1* gene is located on chromosome 1 at locus p34.2. The *SLC2A1* gene has 10 exons and 3 putative enhancers. The rs841847 polymorphism (C > T) is located in the insulin-responsive enhancer-2 sequence within intron 2, suggesting a gene expression-modifying effect [28]. Single nucleotide variants within the *SLC2A1* gene, including the rs841847 polymorphism, have been linked to type-2 diabetes mellitus and related conditions, such as diabetic nephropathy in type-1 and type-2 diabetes mellitus, as well as albuminuria [28,29,30,31,32,33,34].

Despite the potentially important role of altered glucose homeostasis in AD, variants of the *SLC2A1* gene have not yet been investigated in AD or in any form of dementia. In the present study, the rs841847 polymorphism was chosen as a candidate risk factor for AD based on its location and its potential regulatory effect on gene expression. We aimed to investigate the possible association between the rs841847 polymorphism and late-onset AD risk, either alone or in genetic interaction with the *APOE* ε4 allele in a well-characterized case–control sample.

## 2. Methods

A total of 743 participants of Hungarian Caucasian origin were enrolled in our case-control study, including 439 patients with late-onset AD (75.6 ± 6.2 years of age (mean ± SD), men 35.2%) and 304 cognitively healthy, elderly control individuals (75.7 ± 5.6 years of age (mean ± SD), men 36.1%). All methods were carried out in accordance with institutional guidelines and regulations, and in line with the Declaration of Helsinki. Participant recruitment and all study protocols were conducted with the approval of the Ethics Committee of the Hungarian Medical Research Council (ETT-TUKEB). Written informed consent was obtained from all participants or their legal representatives.

An exclusion criterion for subject recruitment was the diagnosis of type-1 or type-2 diabetes mellitus, assessed through comprehensive clinical evaluations. These evaluations included comprehensive laboratory tests such as fasting blood glucose levels and a detailed review of the participants’ medical history. The AD patients with a minimum age at onset of 65 years were recruited from the Memory Clinic of the Department of Psychiatry, University of Szeged, Hungary. The diagnoses of late-onset AD were established by experienced neurologists and psychiatrists according to the National Institute of Neurological and Communicative Disorders and Stroke/Alzheimer’s Disease and Related Disorders Association (NINCDS/ADRDA) criteria [35].

Control subjects were recruited from the same geographical area as AD patients and had no history of neurological or psychiatric disorders. The Mini-Mental State Examination (MMSE) was applied to measure global cognitive performance: the mean MMSE score in the AD group was 17.5 ± 5.6 (mean ± SD), while in the control group, MMSE scores were equal to or higher than 28 points and none of the control individuals had any verified symptoms of dementia. The clinical evaluation of all study participants was performed without prior knowledge of genetic background, and genotyping was carried out blinded to the clinical status of the subjects. Peripheral blood samples were collected and processed using standard protocols in accordance with the relevant guidelines and regulations.

Genomic DNA was extracted from whole blood using the Roche High Pure PCR Template Preparation Kit according to the manufacturer’s instructions (Roche Holding AG, Basel, Switzerland). Following enzymatic lysis of the cells, nucleic acids were captured on a silica column, then purified via washing steps and eluted with low-salt buffer. All buffers and reagents were supplied by the manufacturer. The DNA was used immediately or stored at −20 °C for later analysis. DNA concentration and purity were assessed using a NanoDrop spectrophotometer (MaestroGen, Taipei, Taiwan).

Genotyping experiments were carried out using fluorescence-labeled TaqMan allelic discrimination assays and quantitative polymerase chain reaction (qPCR) analysis with premade, commercially available assay mixes (Thermo Fisher Scientific, Waltham, MA, USA) and master mix (Thermo Fisher Scientific, Waltham, MA, USA). Characteristics of the investigated polymorphisms and the assay identification numbers are summarized in Table 1. The qPCR amplification was conducted in single-plex reactions in 96-well plates with a total volume of 20 μL using the following amplification protocol: 95 °C for 10 min, 40 cycles of 92 °C for 15 s and 60 °C for 1 min. Fluorescence measurements were performed using the CFX96 Touch Real-Time PCR Detection System (Bio-Rad Laboratories, Inc., Hercules, CA, USA).

Categorical variables, including gender, allele and genotype frequencies, were analyzed by Fisher’s exact and Pearson’s χ^2^-tests, while continuous parameters such as age and MMSE values were compared by *t*-test for independent samples. Hardy-Weinberg equilibrium (HWE) was tested by Pearson’s χ^2^-test (Supplementary Table S1).

In the control group, genotype distributions were consistent with HWE for both *APOE* rs429358 (χ^2^ = 1.832, df = 2, *p* = 0.40) and rs7412 polymorphisms (χ^2^ = 5.167, df = 2, *p* = 0.075). Similarly, in the AD group, no deviation from HWE was detected for *APOE* rs429358 (χ^2^ = 0.227, df = 2, *p* = 0.89) or rs7412 polymorphisms (χ^2^ = 0.512, df = 2, *p* = 0.78). The *SLC2A1* rs841847 polymorphism also conformed to HWE in both the control (χ^2^ = 0.747, df = 2, *p* = 0.69) and AD groups (χ^2^ = 0.764, df = 2, *p* = 0.68). These findings indicate no evidence of genotype distribution distortion and support the reliability of the genotyping procedures. Furthermore, the results do not suggest any HWE-related signal of phenotype misclassification within the analyzed cohorts.

Minor allele frequencies (MAF) of the investigated polymorphisms are presented in Table 1. MAFs of the Hungarian control study population were calculated from genotyped data. Reference MAF values for European and global populations were retrieved from the Genome Aggregation Database (gnomAD v4.0). The observed allele frequencies in our cohort were consistent with those reported in both global and European reference populations.

A multivariate logistic regression model, including age, sex, and *APOE* ε4 status (ε4 allele carriers vs. non-carriers) as simultaneous covariates, was defined as the primary analytical framework for testing the association between *SLC2A1* rs841847 and AD risk. Analyses were performed using the forced-entry method. The primary genetic model assumed a T allele carrier effect (C/T + T/T vs. C/C genotype) on AD risk based on prior functional evidence suggesting allele-dependent effect of the T allele on *SLC2A1* expression and the relatively low frequency of the T/T genotype.

Additional genetic models, including genotypic and allelic models, were conducted as secondary exploratory analyses to evaluate the consistency of the primary association across alternative genetic coding schemes and potential effect modifiers. Interaction effects between *SLC2A1* rs841847 and *APOE* ε4 status, as well as between *SLC2A1* rs841847 and sex, were assessed in separate multivariable logistic regression models by including multiplicative interaction terms (rs841847 × *APOE* ε4 and rs841847 × sex). Sex-stratified analyses were additionally performed for descriptive purposes (Supplementary Table S2). Exploratory multivariable logistic regression analyses were conducted separately in males and females under a genetic model of T+ vs. C/C genotype, adjusted for age and *APOE* ε4 status. Analyses were performed using the forced-entry method.

The primary analysis was defined a priori and not adjusted for multiple comparisons. Secondary genetic models, interaction analyses, and sex-stratified analyses were subjected to false discovery rate (FDR) correction using the Benjamini–Hochberg procedure, with q < 0.05 considered statistically significant. Effect sizes were expressed as odds ratios (OR) with 95% confidence intervals (CI).

## 3. Results

Demographic characteristics and *APOE* genotype frequencies of the investigated groups, including 439 AD patients and 304 cognitively healthy elderly controls, are summarized in Table 2. Regarding the demographic and background parameters of the examined samples, no significant differences were found between the AD cases and controls in mean age or in the male to female ratio; whereas MMSE mean scores and *APOE* genotype and allele frequencies were found to differ significantly.

The *APOE* ε3/ε4 and ε4/ε4 genotypes were significantly over-represented in the AD as compared to the control group (ε3/ε4 genotype: AD: 39.2%, control: 17.8%; ε4/ε4 genotype: AD: 7.5%, control: 0.3%; χ^2^ = 76.536, df = 5, *p* < 0.001). The *APOE* allele frequencies also differed markedly between cases and controls. Although the ε3 allele was the most prevalent allele in both investigated groups, it was significantly less frequent among AD patients relative to the controls (AD: 68.5%; control: 82.9%). On the other hand, the ε4 allele occurred with a significantly higher frequency in the AD compared to the control group (AD: 28.3%; control: 10.2%). The ε2 allele was the least common allele in both cases and controls (AD: 3.3%; control: 6.9%). These differences in allele distribution were statistically highly significant (χ^2^ = 75.945, df = 2, *p* < 0.0001), which confirms a strong association between *APOE* allele status and disease status.

Accordingly, the ε4 allele carriers had a significantly increased risk for AD (OR: 3.903; 95% CI: 2.782–5.477; *p* < 0.001). When examining the effect of ε4 allele dosage, heterozygous individuals carrying one copy of the ε4 allele (ε3/ε4 or ε2/ε4) exhibited a moderately increased risk for AD (OR = 3.291; 95% CI: 2.334–4.640; *p* < 0.001), whereas homozygous carriers of ε4 allele (ε4/ε4) demonstrated a substantially higher risk (OR = 35.799; 95% CI: 4.856–263.915; *p* < 0.001). The wide confidence interval for ε4 homozygotes reflects the very low frequency of this genotype in the control group, which may result in unstable risk estimates. The corresponding odds ratio should be interpreted with caution and is presented as an exploratory, descriptive estimate. Our findings corroborate previous reports on the dose-dependent relationship between ε4 allele number and AD susceptibility. In contrast, carriers of the ε2 allele, either as ε2/ε2 or ε2/ε3 genotypes, appeared to have a lower prevalence among AD patients, suggesting a potential protective effect, although the small number of ε2 homozygotes limits definitive conclusions.

Genotype distribution of the investigated *SLC2A1* rs841847 polymorphism is presented in Table 3. In the primary analysis, a multivariate logistic regression model adjusted for age, sex, and *APOE* ε4 status (ε4 allele carriers versus non-carriers) demonstrated that carriers of the rs841847 T allele (C/T + T/T genotypes) had a significantly reduced risk for AD compared to C/C homozygotes (OR = 0.672; 95% CI: 0.493–0.916; *p* = 0.012) (Table 4).

Secondary exploratory analyses included genotypic and allelic models. The genotype distribution differed between cases and controls with higher C/C genotype frequency in the AD compared to the control group (AD: 60.4%, control: 50.7%; χ^2^ = 6.941, df = 2, *p* = 0.031). However, this association did not remain statistically significant after FDR correction (q = 0.062). Allele frequency distributions showed a nominal association with AD (C allele: AD: 77.2%; control: 71.9%; T allele: AD: 22.8%, control: 28.1%; Fisher’s exact test: *p* = 0.021), which also did not remain significant after correction for multiple testing by FDR (q = 0.062).

Interaction analyses revealed no significant epistasis between *APOE* ε4 status and the *SLC2A1* rs841847 polymorphism on AD risk (*p* = 0.104; FDR correction: q = 0.156). Similarly, no significant interaction between *SLC2A1* rs841847 polymorphism and sex was detected (*p* = 0.339; FDR correction: q = 0.339). Sex-stratified analyses (Supplementary Table S2) showed a significant association in males (*p* = 0.007; FDR correction: q = 0.042), whereas no significant association was observed in females (*p* = 0.239; FDR correction: q = 0.287). However, given the absence of a significant *SLC2A1* rs841847 × sex interaction, our data do not support a statistically significant sex-dependent effect of the polymorphism. Hence, sex-stratified analyses should be interpreted with caution and are presented as exploratory, descriptive results.

## 4. Discussion

The present case-control association study demonstrated *SLC2A1* rs841847 polymorphism as a potential candidate SNP for further examination as a predisposing genetic factor for AD. We found that the minor T allele-containing genotypes were significantly over-represented in controls compared to the AD cases. Hence, according to our data, the presence of the variant T allele significantly decreased the susceptibility to late-onset AD.

Our results support the role of the *APOE* ε4 allele in late-onset AD. The observed *APOE* allele frequencies in the investigated Hungarian sample are consistent with findings of numerous studies across diverse populations [10,11]. This concordance with international data strengthens the validity of our cohort, the reliability of the genotyping data and provides a robust framework for examining additional candidate risk factors, such as the *SLC2A1* rs841847 polymorphism, in relation to AD susceptibility.

Despite the lack of statistically significant interaction between the *SLC2A1* rs841847 C/C and *APOE* ε4 allele-containing genotypes in our data, several lines of evidence from the broader literature support the biological plausibility of potential interactions between glucose transport dysregulation and apolipoprotein E-mediated pathways in AD [9]. *APOE* ε4 carriers, regardless of dementia status, exhibit reduced cerebral glucose metabolism compared to non-carriers in brain regions commonly affected in AD [36,37].

Preclinical studies using *APOE* gene-targeted replacement mice support these clinical observations, showing apolipoprotein E isoforms affected brain glucose metabolism and insulin signaling differently. *APOE* gene-targeted replacement mice are genetically engineered animal models in which the endogenous murine Apoe gene is replaced by human *APOE* gene variants (e.g., ε2, ε3 or ε4). This gene modification allows the study of the isoform-specific effects of apolipoprotein E under physiological conditions. In particular, *APOE* ε4-targeted replacement mice exhibit altered regulation of glucose uptake and metabolism compared to *APOE* ε3-targeted replacement mice [38]. Although we failed to find a statistically significant interaction between the *SLC2A1* rs841847 C/C and *APOE* ε4 allele-containing genotypes in our sample, it does not rule out the existence of a biologically meaningful interplay. The limited sample size and the low frequency of certain genotype combinations reduce the power to detect subtle effects and gene–gene interactions. However, larger cohorts may reveal interactions undetectable in moderate case–control studies.

The strong epidemiological associations between AD and type-2 diabetes mellitus suggest the existence of common pathophysiological processes, including cerebrovascular alterations, insulin resistance, chronic hyperglycemia, and recurrent hypoglycemic episodes [18]. Type-2 diabetes mellitus is one of the most important risk factors for developing AD. Furthermore, persons with type-2 diabetes mellitus have a higher incidence of cognitive decline and AD [39,40,41]. Gene clustering and functional evaluation of differentially expressed genes in AD and type-2 diabetes identified shared pathways and correlations between pathogenic genes, including glucose metabolism-related genes such as glucose transporter 2 [19,42].

GLUT1, encoded by the *SLC2A1* gene, is a highly abundant membrane protein in human red blood cells, and is also prominently expressed in blood–brain barrier endothelial cells, astrocytes, and other metabolically active tissues. Its expression is regulated in a tissue-dependent manner by factors such as glucose availability, hypoxia, and hormonal signals, including insulin [21]. GLUT1 plays a key role in regulating the rate of cerebral glucose uptake at the blood–brain barrier [18]. Downregulation of GLUT1 levels at the blood–brain barrier was demonstrated in AD, which correlated with concomitant impairment in glucose transport that may contribute to neuronal energy deficits and neurodegeneration [24]. The investigated candidate *SLC2A1* gene in the present study was selected based on its pivotal role in glucose metabolism, the dysregulation of which has been implicated in both AD and type-2 diabetes mellitus.

To our knowledge, this is the first study that addressed the rs841847 polymorphism or any other *SLC2A1* gene variants regarding AD susceptibility; however, *SLC2A1* polymorphisms were extensively studied for population distributions and different disease associations [28,29,30,31,32,33]. A systematic meta-analysis of case–control association studies demonstrated a clear association between the rs841847 polymorphism and diabetic nephropathy in type-2 diabetes mellitus in a recessive model [30]. A more recent, comprehensive meta-analysis confirmed these findings in type-2 diabetes mellitus, both with and without diabetic nephropathy [33].

In the *SLC2A1* gene, three putative enhancer regions were identified by sequence similarity to known enhancer regions of the mouse and rat genes. Enhancer-1 and enhancer-3 sequences are located upstream of exon 1, while the enhancer-2 element is positioned within intron 2 [28]. The rs841847 polymorphism seems to have functional consequences and could be a contributing factor influencing genetic susceptibility to AD as it is located in the putative enhancer-2 sequence that has the potential to modulate gene expression [28]. Additionally, the rs841847 polymorphism resides within a predicted binding site for upstream stimulatory factor (USF), which is a transcription factor that confers responsiveness to stimulation by insulin and glucose [43,44].

Indeed, it has been demonstrated that the rs841847 polymorphism can significantly modify the tissue-dependent expression of the GLUT1 protein. In the presence of the rs841847 variant allele, significantly decreased expression levels of the GLUT1 membrane protein were measured in erythroid cells by antibody-based flow cytometry [45]. Consistent with this result, the mutant allele significantly reduced enhancer-driven transcriptional activity in both HEK-293T and HepG2 cell lines measured by luciferase reporter assays [45]. These findings support the hypothesis that the rs841847 polymorphism alters the enhancer activity of the *SLC2A1* gene and indicates a direct functional effect on gene regulation. Bioinformatic analyses provide a plausible explanation for the reduced expression by indicating that this variant disrupts multiple transcription factor binding sites, including ARNT2 (aryl hydrocarbon receptor nuclear translocator 2), which is potentially related to hypoxia-responsive pathways [45].

The consistency across erythrocyte membrane protein levels, in vitro reporter assay results, and bioinformatic findings support the hypothesis that the rs841847 polymorphism exerts a biologically relevant regulatory effect on *SLC2A1* gene expression. A previous study reported elevated GLUT1 expression levels in erythrocytes of AD patients compared to controls [25]. Our findings demonstrated a higher frequency of the rs841847 C/C genotype in the AD group, which has previously been associated with higher erythrocyte GLUT1 expression levels.

These findings should be interpreted cautiously, since no direct functional validation is currently available in blood–brain barrier endothelial cells or neuronal tissues. Given the tissue-specific regulation of *SLC2A1* gene expression, observations derived from peripheral blood cells cannot be directly extrapolated to changes in cerebral glucose transport mechanisms. The increased erythrocyte GLUT1 expression levels reported in AD and the reduced GLUT1 expression levels observed at the blood–brain barrier in AD may reflect different underlying regulatory mechanisms. These findings suggest that rs841847-related regulatory effects may differ across tissues, and therefore, the observed peripheral effects cannot be assumed to directly reflect the regulation of GLUT1 levels at the blood–brain barrier.

Although the observed genetic association and peripheral biomarker findings are directionally consistent, the present study does not establish a direct mechanistic link between rs841847 and cerebral glucose transport dysfunction. Further studies involving brain-specific functional analyses and tissue-specific expression quantitative trait locus (eQTL) investigations are required to clarify the functional relevance of this variant in the central nervous system. Accordingly, the observed association should be interpreted as a statistical association that may reflect complex, tissue-dependent regulatory effects of *SLC2A1* rs841847 polymorphism, contributing to AD susceptibility.

## 5. Conclusions

The present study demonstrates an association between the *SLC2A1* rs841847 polymorphism and reduced risk for AD in a Hungarian case–control cohort, suggesting a potential protective effect of the minor T allele.

Evidence from erythrocyte-based assays, in vitro reporter systems, and in silico analyses suggests that rs841847 exerts regulatory effects on *SLC2A1* gene expression; however, these findings cannot be directly extrapolated to the blood–brain barrier or neuronal glucose transport. Therefore, the functional interpretation of these results in the central nervous system remains limited.

Taken together, these results demonstrate *SLC2A1* rs841847 polymorphism as a potential genetic modifier of AD susceptibility and provide a rationale for further replication studies in independent cohorts and functional investigation in brain-relevant experimental systems.

## 6. Limitations

The following limitations should be considered when interpreting the present findings. AD diagnosis was based on NINCDS–ADRDA clinical criteria without biomarker confirmation, which may have resulted in potential disease misclassification. Non-differential phenotypic misclassification inherent to the use of clinical-criteria-only diagnosis would generally be expected to bias effect estimates toward the null, suggesting that the observed odds ratio may represent a conservative estimate [46]. In addition, control subjects were classified using MMSE scores without a full neuropsychological assessment; therefore, the inclusion of individuals with prodromal cognitive impairment cannot be fully excluded. A limitation of the present study is that several potentially relevant confounding factors, including vascular comorbidities, body mass index, insulin resistance markers, education level, and medication use, were not available and could not be included in the multivariate analysis. Accordingly, residual confounding cannot be completely excluded.

Principal component analysis and genomic inflation factor (λ) estimation were not feasible due to the absence of genome-wide genotype data. Although all participants were unrelated Hungarian individuals recruited from a single geographic region and clinical center, population stratification cannot be completely excluded. The lack of an independent replication cohort further limits the robustness and generalizability of the observed association. Moreover, linkage disequilibrium and haplotype structure at the *SLC2A1* locus were not evaluated; therefore, it cannot be excluded that the observed association of rs841847 reflects linkage with another common variant in the region.

Finally, the moderate sample size limited statistical power for detecting modest interaction effects and for stable estimation in less frequent genotype groups. Accordingly, the absence of significant interaction effects should be interpreted cautiously. Further studies in larger independent cohorts and more detailed investigations of the *SLC2A1* locus are needed to further elucidate its potential contribution to AD and to provide convergent support.

## Figures and Tables

**Table 1:** Key data of the examined SNPs.

Gene (MIM#)	Polymorphism (Assay ID)	Position	Alleles (wt > mt)	Location	Amino Acid Change	MAF Reference Total	MAF Reference European	MAF Study Cohort
*SLC2A1* (138140)	rs841847 (C_1166180_10)	chr1:42937037	C > T	Intron 2 (Enh-2)	-	0.188	0.249	0.281
*APOE* (107741)	rs429358 (C_3084793_20)	chr19:44908684	T > C	Exon 4	Cys112Arg	0.149	0.151	0.102
*APOE* (107741)	rs7412 (C_904973_10)	chr19:44908822	C > T	Exon 4	Arg158Cys	0.078	0.074	0.069

*SLC2A1*: solute carrier family 2 member 1; *APOE*: apolipoprotein E; Assay ID indicates the specific pre-designed TaqMan SNP assays by Thermo Fisher Scientific (Waltham, MA, USA); wt: wild-type allele; mt: mutant allele; Enh-2: enhancer 2; MAF: Minor Allele Frequency; MAF Reference Total: global MAF reported in Genome Aggregation Database (gnomAD) v4.0 across all populations; MAF Reference European: MAF reported in the European ancestry population in gnomAD v4.0; MAF Study cohort: MAF observed in the Hungarian study control population.

**Table 2:** Demographic characteristics and *APOE* genotype frequencies of the investigated groups.

	AD Patients *n* = 439	Controls *n* = 304
Age (years; mean ± SD)	75.6 ± 6.2	75.7 ± 5.7
Sex (male/female (%))	35.2/64.8	36.1/63.9
MMSE (scores; mean ± SD)	17.5 ± 5.6	≥28
*APOE* genotypes		
ε2/ε2 *n* (%)	0 (0%)	4 (1.3%)
ε2/ε3 *n* (%)	19 (4.3%)	28 (9.2%)
ε2/ε4 *n* (%)	10 (2.3%)	6 (2.0%)
ε3/ε3 *n* (%)	205 (46.7%)	211 (69.4%)
ε3/ε4 *n* (%)	172 (39.2%)	54 (17.8%)
ε4/ε4 *n* (%)	33 (7.5%)	1 (0.3%)

AD: Alzheimer’s disease, MMSE: Mini Mental State Examination, *APOE* genotypes: apolipoprotein E genotypes defined by rs429358 and rs7412 polymorphisms.

**Table 3:** Genotype frequencies of the *SLC2A1* rs841847 polymorphism.

*SLC2A1* Genotypes rs841847	AD Patients *n* = 439	Controls *n* = 304
C/C *n* (%)	265 (60.4%)	154 (50.7%)
C/T *n* (%)	148 (33.7%)	129 (42.4%)
T/T *n* (%)	26 (5.9%)	21 (6.9%)

AD: Alzheimer’s disease, *SLC2A1*: solute carrier family 2 member 1.

**Table 4:** Multivariate logistic regression analysis of the *SLC2A1* rs841847 polymorphism (T+ vs. C/C genotypes) and AD risk adjusted for age, sex, and *APOE* ε4 status.

Variables	OR	95% CI	*p*-Value
*SLC2A1* rs841847 (T+ vs. C/C)	0.672	0.493–0.916	0.012
Age	1.003	0.978–1.030	0.794
Sex (female vs. male)	0.942	0.682–1.301	0.717
*APOE* ε4 status (ε4+ vs. ε4−)	3.921	2.788–5.513	<0.001

AD: Alzheimer’s disease; OR: odds ratio; 95% CI: 95% Confidence Intervals; *SLC2A1*: solute carrier family 2 member 1; *APOE*: apolipoprotein E; *APOE* ε4 status: the presence or absence of the *APOE* ε4 allele defined by rs429358 and rs7412 polymorphisms.

## Data Availability

The datasets generated and analyzed during the current study are publicly available in the Zenodo repository. The data can be accessed at https://zenodo.org/records/16569942 (accessed on 25 May 2026) (DOI: 10.5281/zenodo.16569942).

## Supplementary Materials

The following supporting information can be downloaded at: https://www.mdpi.com/article/10.3390/biom16060808/s1, Table S1: Hardy-Weinberg equilibrium analyses of the examined SNPs in AD cases and controls; Table S2: Sex-stratified multivariate logistic regression analysis of the *SLC2A1* rs841847 polymorphism (T+ vs. C/C genotypes) and AD risk adjusted for age and *APOE* ε4 status.
