# Supplementary material for: The Variant T Allele of SLC2A1 rs841847 Confers Moderate Protection Against Late-Onset Alzheimer’s Disease
Source: Biomolecules. 2026 May 29;16(6):808. doi: 10.3390/biom16060808 (PMC13297088; doi:10.3390/biom16060808)
Supplement: Supplementary file 1 [file biomolecules-16-00808-s001.zip › biomolecules-4295751-supplementary.pdf]

Supplementary Table S1. Hardy-Weinberg equilibrium analyses of the examined SNPs in AD cases and controls.

Gene	SNP	Genotypes (n)			χ^2	df	*p*-value
SLC2A1	rs841847	C/C	C/T	T/T			
	AD (n=439)	265	148	26	0.764	2	0.68
	Controls (n=304)	154	129	21	0.747	2	0.69
APOE	rs429358	T/T	T/C	C/C			
	AD(n=439)	224	182	33	0.227	2	0.89
	Controls (n=304)	243	60	1	1.832	2	0.40
APOE	rs7412	C/C	C/T	T/T			
	AD(n=439)	410	29	0	0.512	2	0.69
	Controls (n=304)	266	34	4	5.167	2	0.075

SNP: single nucleotide polymorphism; AD: Alzheimer's disease; *SLC2A1*: solute carrier family 2 member 1; *APOE*: apolipoprotein E.

Supplementary Table S2. Sex-stratified multivariate logistic regression analysis of the *SLC2A1* rs841847 polymorphism (T+ vs. C/C genotypes) and AD risk adjusted for age and *APOE* ε4 status.

Sex	rs841847 genotypes	AD (n)	Controls (n)	OR	95% CI	*p*-value
Male		154	107	0.475	0.278-0.814	0.007
	C/C	93	49			
	C/T	49	51			
	T/T	12	7			
Female		285	197	0.795	0.542-1.165	0.239
	C/C	172	105			
	C/T	99	78			
	T/T	14	14			

AD: Alzheimer's disease; OR: odds ratio; 95% CI: 95% Confidence Intervals; *SLC2A1*: solute carrier family 2 member 1; *APOE*: apolipoprotein E; *APOE* ε4 status: the presence or absence of the *APOE* ε4 allele defined by rs429358 and rs7412 polymorphisms.
